# Investigating energy literacy and its structural model for citizens of Mashhad

**DOI:** 10.1016/j.heliyon.2022.e11449

**Published:** 2022-11-05

**Authors:** Hamed Sayarkhalaj, Majid Fatemi Khesal

**Affiliations:** a University of Isfahan, Isfahan, Iran; b Ferdowsi University of Mashhad, Mashhad, Iran

**Keywords:** Attitude, Energy literacy, Environment, Climate change, Knowledge

## Abstract

Today, one of the most crucial environmental problems is energy consumption. Excessive energy consumption has led to environmental damage such as climate change. Energy and climate change are interrelated. Energy literacy is one of the tools to achieve energy sustainability. Energy literacy can reduce energy consumption by citizens. This research aimed to a sociological analysis of energy literacy among the citizens of Mashhad. The research method is a survey in which 384 citizens of Mashhad were selected based on a multi-stage cluster sampling method and data gathered by a questionnaire. The results showed that knowledge of energy consumption and attitude and effectiveness towards energy have a significant and direct relationship with energy consumption behavior. The attitude and effectiveness towards energy have a significant and direct relationship with the knowledge of energy consumption. Estimating the goodness of fit indices and the structural and measurement coefficients confirmed the model. Teaching proper behavioral patterns in energy consumption at the family and school can effectively improve citizens' energy literacy.

## 1 Introduction

Today there are many environmental problems at the global level. These problems are caused by the environmental impacts of various sectors such as shipbuilding and marine transportation, industrial products such as steel and metallurgy, and the construction industry, which causes an increase in greenhouse gases and climate change (Dulebentes, 2018; Abioye et al., 2019; Liang et al., 2021; Zhang et al., 2021; Pasha et al., 2021; Izydorczyk et al., 2021). One of the most crucial environmental problems is energy consumption. Excessive energy consumption has led to environmental damage such as climate change. Energy and climate change are interrelated (Van Ruijven et al., 2019); Increasing energy consumption leads to greenhouse gas emissions and climate change (Banerjee et al., 2021); On the other hand, the continuation of climate change increases energy consumption (De Cian and Sue Wing, 2019). Statistics show that countries' overall performance is red in greenhouse gas emissions, renewable energy, energy consumption, and climate policy (CCPI, 2022).

In the present century, issues such as energy resources, energy consumption, and energy dependence have become important because of the connection between human life and energy (DeWaters and Powers, 2011a). Today, researchers recognize energy literacy as one of the most effective tools to achieve energy sustainability (Lee et al., 2019). Energy use is a significant challenge that requires awareness and adaptation to social levels. Energy literacy generally includes knowledge and understanding of energy, attitudes, values, intentions, and appropriate behaviors. Energy literacy informs people and makes them aware of their responsibilities toward energy consumption (Setyowati et al., 2019; Cotton et al., 2021). People with energy literacy have a basic understanding of energy consumption and understand the environment and the effects of energy production and consumption. They also identify the effects of energy-related actions on individuals, companies, and groups worldwide. Energy literacy plays an essential role in developing societies; Because energy-conscious citizens are futuristic and can think of alternative energies (DeWaters and Powers, 2013). Citizens with energy literacy will equip the ability to make thoughtful and responsible decisions and actions related to energy. Energy literacy in the modern world is one of the most crucial life skills that empower citizens (DeWaters and Powers, 2011b).

Modern and densely populated cities are on the rise; probably by 2050, more than 80% of the population will live in cities. Thus, cities face many environmental challenges, such as energy consumption (UNEP, 2012; Cepeliauskaite and Stasiskiene, 2020). So it is natural that energy literacy in urban society also becomes an important issue (Chodkowska-Miszczuk et al., 2021). Despite the importance of energy literacy, few studies on this concept among citizens. Most studies have examined energy literacy among students, While households consume more than 30% of energy (Cotton et al., 2021). Citizens consume energy in various sectors, and their skills, perceptions, and attitudes towards energy are directly related to reducing or increasing their energy footprint (Bloom and Quebec Fuentes, 2019). Based on the research, citizens consider energy saving the most crucial strategy to reduce environmental impacts. However, currently, they do not use energy efficiently and have an energy efficiency gap (Lindén et al., 2006; Semenza et al., 2008; Allcott and Greenstone, 2012). Therefore, the problem of energy literacy is not only relevant to students and should also be considered among citizens.

The issue of energy literacy in Iran has also received less attention. There are worrying statistics about increasing energy consumption in Iran. According to the EIA, Iran is the largest energy consumer in the Middle East. The highest consumption of fossil fuels such as gas is in the residential sector with 35% (IEA, 2019). So far, energy policymakers in Iran have implemented various policies, such as increasing the market price of energy carriers; But these policies have not been very effective. Therefore, behavioral strategies to change energy consumption patterns seem more effective (Hamidi Razi et al., 2021).

Energy literacy level is the research problem. Energy literacy is critical in metropolitan areas; Because large populations live in dense spaces, and non-compliance with environmental issues has a much more significant impact on these cities. Mashhad is the second largest metropolis in Iran and has a population of about three million. The metropolis hosts an estimated 29 million tourists annually. Therefore, it has a large amount of energy consumption. Examining the various dimensions of energy literacy in this city makes it possible to provide solutions and strategies related to energy consumption. The main objective of this research is to investigate energy literacy among the citizens of Mashhad. The critical questions of this research are:

• What is the performance of citizens in energy literacy in the components of knowledge, attitude, and behavior?
• What are the relationship between knowledge, attitude, and behavior?

## 2 Literature review

Energy literacy is closely related to environmental literacy; Because one of the most important aspects of the environment is the energy debate. Therefore, this model can be generalized to energy literacy (Schultz, 2002). The traditional linear model of responsible environmental behavior shows that increasing knowledge leads to environmental attitudes, making positive environmental behavior possible (Hungerford and Volk, 1990).

Frick et al. (2004) have proposed a threefold classification of environmental knowledge: system knowledge, action-related knowledge, and effectiveness knowledge. System knowledge is related to the knowledge of environmental problems and their causes. Action-related knowledge is individuals' awareness of behavioral measures to reduce environmental problems; Knowledge of effectiveness also requires the ability to effectively reduce environmental problems. They argue that knowledge related to action and knowledge of effectiveness directly predict protective behaviors, while two other types of environmental knowledge indirectly influence system knowledge. Pearson and Young (2002) argue that energy literacy has three dimensions: the cognitive or knowledge dimension, which includes understanding skills; the effectiveness dimension, which includes energy-sensitive attitudes; and the behavioral dimension, which includes intentional energy behaviors.

In the theory of planned behavior, a person’s behavior can be positive or negative and influenced by attitude (Ajzen, 1991). According to this theory of energy consumption behavior, if a person has a positive attitude towards energy conservation, he will show a high level of energy conservation behavior (Van den Broek, 2016). Attitudes towards energy can motivate energy consumption behavior and play a significant role in energy consumption behavior (Stern, 2000). Past research has shown that the difference between energy conservation attitudes and behaviors is high (Viklund, 2004).

Energy literacy and the relationship between its constructs have been analyzed in various studies. However, most of these studies have been done on a student, while citizens have received less attention. The findings of DeWaters and Powers (2008) showed that students could only recognize energy problems and lacked the knowledge and ability to create solutions. DeWaters and Powers conducted two other studies in 2011 on students' energy literacy. The first study (2011a) concluded that students were merely concerned with energy-related problems while lacking the necessary knowledge and skills. The second study results (2011b) also showed that students think more about energy consumption after completing a project-based energy curriculum; They also put more effort into consuming energy.

Bodzin et al. (2013) concluded in their research that the geographical curriculum approach could enhance knowledge of energy acquisition, energy production, storage and transportation, and energy consumption and conservation. In their research, Lee et al. (2015) concluded a significant and direct relationship between the dimensions of effectiveness, knowledge, and energy consumption behavior. The findings of Akitsu et al. (2017) showed that the dimensions of energy literacy, including knowledge, attitude, and behavior, have a significant and direct relationship. Research results Aguirre-Bielschowsky et al. (2017) showed that most children had a positive attitude toward energy conservation, While they did not intend to save energy. In their research, Chiu and DeWaters (2018) showed that attitudes towards energy have a moderate correlation with energy efficiency and behavior and a weak correlation with energy knowledge. The era of Covid-19 also greatly affected energy and showed that energy-related actions and behaviors related to energy have essential effects on energy resources. Travel bans and border closures reduced fuel consumption; Also, and the demand for renewable energy increased (Madurai Elavarasan et al., 2020; Khan et al., 2020). In their research, Wang and Su (2020) showed that measures such as reducing economic activities and traffic restrictions during the Covid-19 era reduce energy consumption and prevent environmental pollution.

Cotton et al. (2021) compared energy literacy between British and Chinese citizens. Their findings showed a significant difference between the knowledge, attitude, and behavioral intentions of the citizens of the two countries; British citizens showed a more positive attitude towards energy conservation than Chinese citizens. On the other hand, Chinese citizens showed higher trust in the government and businesses to take action on energy issues. In another study, Ilham et al. (2022) concluded that students have an acceptable level of positive attitude and practice toward energy conservation. Also, the findings of Lee et al. (2022) showed that the energy knowledge of 12th-grade students was low, and the perceived values, attitudes, intentions, and behaviors related to energy conservation were relatively high. Energy knowledge did not directly affect the intention or behavior of energy conservation, while despite the value and attitude towards it, it could have an indirect effect.

## 3 Research methodology

### 3.1 Research framework

This study examines the relationship between knowledge of energy consumption, attitudes, and effectiveness towards energy with energy consumption behavior in the form of a general hypothesis. The research hypothesis is combined and multivariate in the form of a structural equation model. Instead of presenting the hypotheses separately in this model, the hypotheses are in the form of a model. Based on this, expect energy consumption behavior to function on knowledge of energy consumption and attitude and effectiveness towards energy. Also, the effect of energy knowledge on energy consumption behavior is direct and indirect, and the effect of attitude and effectiveness on energy is direct.

### 3.2 Samples

**Figure 1:**
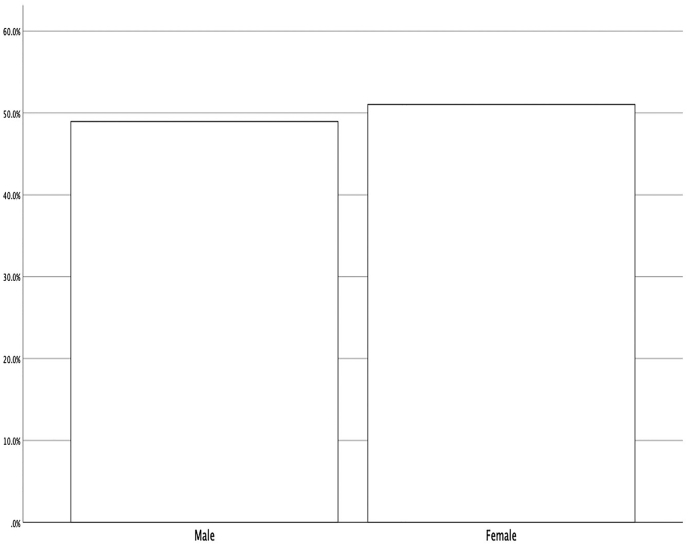
Distribution of respondents by gender.

**Figure 2:**
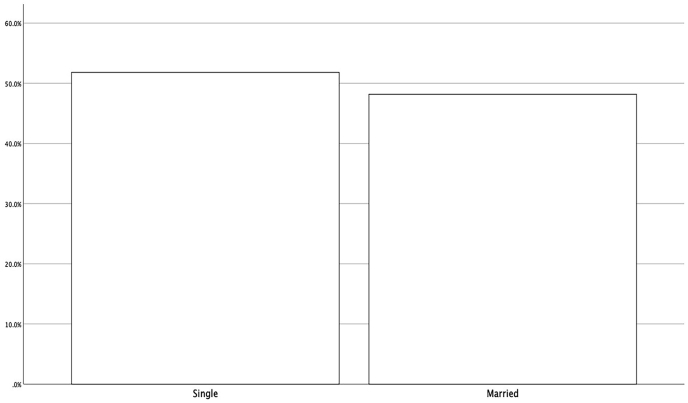
Distribution of respondents by marital status.

**Figure 3:**
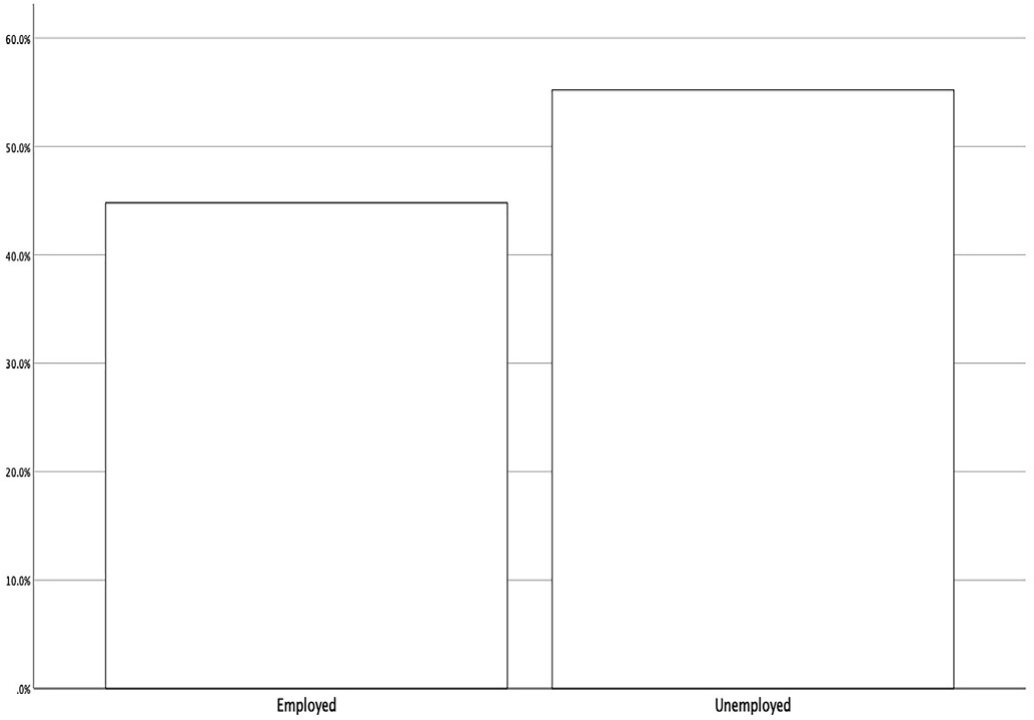
Distribution of respondents by job status.

The statistical population of this study consists of citizens aged 15 years and older living in Mashhad who lived in 12 districts of Mashhad. The sample size is estimated using Cochran’s formula. According to the 95% confidence level, select a sample of 384 people. The sampling method in this study is a multi-stage cluster in which select some neighborhoods have to select respondents. Then, selected neighborhoods, selected blocks are selected, and finally, a sample of citizens is selected, and use the standard method is to select individuals in selected households (see Figures 1, 2 and 3).

### 3.3 Study procedure and instrument

**Figure 4:**
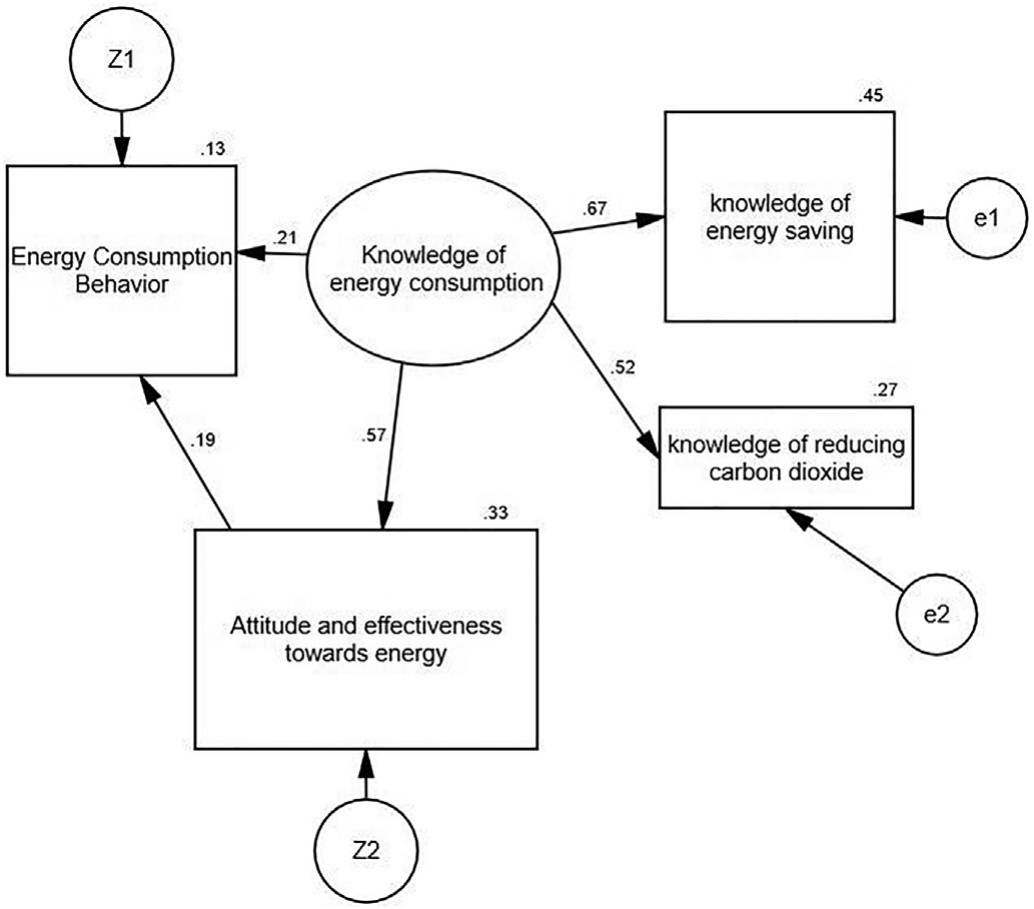
Structural equation model.

The present study uses a qualitative approach and a survey method. Data were analyzed using SPSS version 23, PLS version 3, and AMOS version 22. In light of the structural relationships between knowledge of energy consumption and attitude and effectiveness towards energy that might be influential in forming energy literacy, this study applied structural equation modeling (SEM) as the primary statistical analysis method. In SEM, a theoretical model is a constructed in which an abstract construct or latent variables, such as knowledge of energy consumption or attitude and effectiveness towards energy, is measured by a few indicators, and the interplay among these constructs is estimated. Questionnaire items were divided into three constructs (the one oval and two rectangles): knowledge of energy consumption or attitude and effectiveness towards energy, shown in Figure 4. Each of these constructs is measured by several indicators or measured variables; for example, the construct of knowledge of energy consumption is measured by two indicators, the knowledge of energy saving and the knowledge of reducing carbon dioxide.

The average variance extracted (AVE) is used to measure the convergent validity (Hair et al., 2014); The Fornell and Larker criterion is used to measure divergent validity (Henseler et al., 2015). Cronbach’s alpha was also used to assess the reliability of the questionnaire. The uses following standard questionnaires to measure energy literacy:

1. Energy Consumption Behavior: This dimension of energy literacy considers the action and behavior of the individual in order to conserve energy and energy consumption (DeWaters et al., 2007). The present study used Abu-Ashour's (2018) questionnaire to measure this variable; This dimension had ten questions.
2. Knowledge of energy consumption: This dimension of energy literacy includes the knowledge of understanding skills in energy consumption. The Chiu and DeWaters Questionnaire (2018) was used to measure this variable; This questionnaire had two dimensions knowledge of energy-saving and knowledge of reducing carbon dioxide; Each of these dimensions had four questions.
3. Attitude and effectiveness towards energy: This dimension refers to positive attitudes and sensitivities towards energies such as fossil and modern (Naderi et al., 2017). The Lee and Tanusia (2016) questionnaire measured this variable; The scale had six questions.

Reliability for the energy literacy scale was determined using Cronbach’s alpha. Also, Convergent validity is measured by average variance extracted (AVE) (Henseler et al., 2015; Hair et al., 2014). The Cronbach’s alpha coefficient and composite reliability of 0.5 or above were considered satisfactory. Too, AVE from all constructs exceeded the minimum criterion of 0.5, indicating that the constructs explained a large portion of the variance (see Table 1).

**Table 1:** Convergent Validity and Reliability of the constructs.

Constructs	Cronbach’s Alpha	Average Variance Extracted (AVE)
Energy Consumption Behavior	0.616	0.527
Knowledge of energy consumption	0.619	0.672
Attitude and effectiveness toward energy	0.765	0.514

In their widely cited article on tests to evaluate structural equation models, Fornell and Larcker (1981) suggest that discriminant validity is established if a latent variable accounts for more variance in its associated indicator variables than shares with other constructs in the same model. To satisfy this requirement, each construct’s average variance extracted (AVE) must be compared with its squared correlations with other constructs in the model.

Discriminant validity is showed that using the Fornell-Larcker criterion; it was confirmed when the AVE of a composite construct was higher than the construct’s highest squared correlation with any other composite construct (see Table 2).

**Table 2:** Discriminant Validity (Fornell-Larcker) of the Energy Literacy scale.

Constructs	Energy Consumption Behavior	Knowledge of energy consumption	Attitude and effectiveness toward energy
Energy Consumption Behavior	0.726		
Knowledge of energy consumption	0.253	0.820	
Attitude and effectiveness toward energy	0.428	0.373	0.717

In this research, ethical principles have been observed by the researchers. The ethical principles of the research have been approved by the Research Ethics Committees of the Ferdowsi University of Mashhad. Informed consent from the participants has also been obtained.

## 4 Findings

### 4.1 Descriptive analysis

Descriptive findings show that 49% of respondents are male, 51% are female, 51.8% are single, and 48.2% are married. 44.8% of the respondents are employed, and 55.2% are unemployed (see Table 3, Figure 1, Figure 2, Figure 3).

**Table 3:** Demographic profile of respondents.

Variable	Percentage (%)
*Gander*	
Male	49
Female	51
*Marital status*	
Single	51.8
Married	48.2
*Job*	
employed	44.8
unemployed	55.2

n = 384.

Table 4 indicates that 5.5% of the respondents have knowledge of energy consumption at a very high level, 18% high, 49.2% moderate, 18.5% low, and 8.9% very low; Therefore, most respondents have a moderate level of knowledge of energy consumption. Other findings indicate that 43.2% of respondents have a very high level of attitude and effectiveness towards energy; Also, 30.2% of the respondents have a high level, 21.1% moderate, 3.9% low, and 1.6% very low, have an attitude and effectiveness towards energy; As a result, most respondents have a high attitude and effectiveness towards energy. Other results show that 4.2% of the respondents have a very high level, and 28.6% have a high level of energy consumption behavior; Also, 33.1% of the respondents are moderate, 24.5% low, and 9.6% very low have energy consumption behavior.

**Table 4:** Citizens' performance regarding energy literacy.

Energy Literacy	Very high	High	Moderate	Low	Very Low
Knowledge of energy consumption	5.5	18	49.2	18.5	8.9
Attitude and effectiveness toward energy	43.2	30.2	21.1	3.9	1.6
Energy Consumption Behavior	4.2	28.6	33.1	24.5	9.6

Table 5 shows that 10.9% of the respondents always and 19.3% of them wash their clothes with cold water most time; While 29.4% of respondents sometimes, 26.3% rarely do so, and 14.1% never do so in order to save energy. Findings on hanging clothes outdoors instead of using a dryer show that 20.8% of respondents always, 32.3% most time, 13.5% sometimes, 18% of them rarely do this, and 14.8% of them never do it.

**Table 5:** Percentage distribution of items related to energy consumption behavior.

Row	Energy Consumption Behavior	Always	Most time	Sometimes	Rarely	Never
1	Wash your clothes in cold water	10.9	19.3	29.4	26.3	14.1
2	Hang your clothes out to dry rather than using a dryer	20.8	32.3	13.5	18.5	14.8
3	Turn off the water when brushing your teeth	60.1	25.5	6.8	3.6	4
4	Turn off the water when washing dishes/use a partially filled sink	55.7	26.6	12.2	2.3	3.1
5	Take short showers	25.5	39.1	20.3	13.3	1.8
6	I turn off high-consumption appliances during peak hours	4.9	15.1	25.5	33.9	20.6
7	Adjust your thermostat to use less heating and/or air conditioning	27.6	40.6	26	4.9	0.8
8	Recycle as much as possible	30.7	33.1	21.6	12.8	1.8
9	Use a green bin or compost as much as possible	14.6	16.9	27.3	28.1	13
10	Use a rain barrel to collect rain water to be used outside to water the gardens in lieu of a hose	8.3	20.3	32.6	25	13.8

Other results show that 60.1% of respondents always, 25.5% most time, 6.8% sometimes, 3.6% rarely turn off the water while brushing, and 4% never do that. Findings on the item of Turn off the water when washing dishes show that 55.7% of respondents always, 26.6% most time, 12.2% sometimes, 2.3% Rarely do this, and 3.1% of them never do this in terms of energy consumption. 25.5% of the respondents always, 39.1% most time, 20.3% sometimes, 13.3% rarely take a shower in a short time, and 1.8% never do so.

Findings show that 4.9% of respondents always turn off high-consumption home appliances during peak hours; 15.1% of respondents do it most time, 25.5% sometimes and 33.9% rarely, and 20.6% never turn on their appliances during peak hours. Findings on placing the heater at a low temperature show that 27.6% of the respondents always do it, 40.6% most time, 26% sometimes do it, and 4.9% rarely do it. Moreover, 0.8% of them never do. 30.7% of respondents always recycle their waste; 33.1% of them do it most time, 21.6% of them sometimes, 12.8% of them rarely do it, and 1.8% never do it.

Other findings indicate that 14.6% of respondents always, 16.9% most time, 27.3% sometimes, 28.1% rarely compost their waste, and 13% never do that. 8.3% of respondents always use rainwater to irrigate the garden; 20.3% of them do it most time, 32.6% sometimes and 25% rarely, and 13.8% never do it.

### 4.2 Pearson correlation analysis

According to the Pearson correlation test, which has a significant and direct relationship between knowledge of energy consumption and energy consumption behavior (r = 0.221); Therefore, increases in knowledge of energy consumption improve energy consumption behaviors. There is also a significant and direct relationship between attitudes and effectiveness towards energy and energy consumption behavior (r = 0.312); The higher a person’s attitude and effectiveness towards energy, the better his energy consumption behaviors. Other results indicate a significant and direct relationship between attitudes and effectiveness towards energy and knowledge of energy consumption (r = 0.414); The more knowledge there is towards energy consumption, the higher the attitude and effectiveness towards energy (see Table 6). The results of this research confirm the assumptions of the linear model of responsible environmental behaviors. Knowledge of energy consumption leads to the formation of attitude and effectiveness towards energy, and finally, attitude leads to the creation of energy consumption behavior.

**Table 6:** Correlation matrix.

Variable	1	2	3
(1) Knowledge of energy consumption	1		
(2) Attitude and effectiveness toward energy	.414∗∗	1	
(3) Energy Consumption Behavior	.221∗∗	.312∗∗	1

∗∗p < 0.01.

### 4.3 Structural equation modeling

According to Table 7, model fit indicators show that the model has a relatively good fit. In order to evaluate the fit of the proposed model, we consider several indicators. Since the chi-square is affected by the sample size, the uses relative chi-square index, the Chi-square index is equal to 1.504; This index should be below 5, calculated according to the desired results.

**Table 7:** Results of structural equation model.

Model	CFI	NFI	NNFI	IFI	RMESA	df	p	x^2^
1	0.997	0.991	0.982	0.997	0.036	1	0.000	1.504

Also, the root of the mean square error of the approximation should be less than 0.8, which is equal to 0.036 in the proposed model. The amount of CFI, NNFI, and IFI components should be more than 0.9, which in the model under study is equal to 0.997, 0.982, and 0.997, respectively. Also, the amount of NFI is 0.991, which is considered desirable. According to the indicators and outputs of AMOS, the model has a good fit. Figure 4 shows the structural equation model.

## 5 Conclusion

Today, energy literacy has become one of the most crucial issues in environmental sociology. Environmental sociologists have concluded that to preserve the environment and reduce energy consumption, there must be changes in one’s knowledge and attitude. Therefore, this study was conducted to investigate the level of energy literacy among the citizens of Mashhad.

The results showed that there is a significant and direct relationship between knowledge and energy consumption behavior; This result was consistent with the findings of Lee et al. (2015), Akitsu et al. (2017), and Chiu and Dewaters (2018); Past research has shown that knowledge of energy consumption has a significant relationship with energy consumption behavior; Knowledge is a precondition for behavior. This result is different from the traditional linear model of environmental literacy, which indirectly considers the role of knowledge. In modern models of energy literacy, energy-related knowledge leads to appropriate behaviors in the field of energy consumption. However, in the studies of Dewaters and Powers (2008), (2011a) and Lee et al. (2022), a kind of gap was observed between knowledge and energy consumption behavior. The findings of the present study were not consistent with these studies.

Other findings showed that there is a significant and direct relationship between knowledge of energy consumption and attitude and effectiveness towards energy; This finding is based on the results of research by Akitsu et al. (2017), Lee et al. (2015), Chiu and Dewaters (2018), Ilham et al. (2022) and Lee et al. (2022). Attitude is one of the essential prerequisites for any behavior. Increasing energy knowledge leads to attitudes and effectiveness toward energy; Awareness of energy and its consumption, a person has an effective attitude towards energy and seeks to be more responsible for energy.

Other results showed a significant and direct relationship between attitudes and effectiveness towards energy and energy consumption behavior; the more positive the attitude towards energy, the more responsible behaviors towards energy we see. This result was consistent with the findings of Chen et al. (2015), Lee et al. (2015), Akitsu et al. (2017), Chiu and DeWaters (2018), and Lee et al. (2022). In contrast, the research results, Aguirre-Bielschowsky et al. (2017), were not coordinated. Attitude towards energy, influenced by the knowledge of energy consumption, is one of the essential stimuli in energy consumption behavior. Attitudes towards energy can motivate behaviors in energy consumption, and a positive attitude towards energy is the basis for energy behaviors.

Findings related to energy literacy can be helpful for decision-makers and policymakers in the city of Mashhad and other regions of Iran in the field of energy and its management. In order to improve energy consumption patterns, they can improve people’s attitudes and knowledge towards energy consumption with educational programs. Holding educational workshops in the field of proper energy consumption, teaching in schools and universities, and promoting proper patterns of energy consumption in various media is a step towards improving the level of knowledge and attitude of citizens in the field of energy. According to previous research in environmental and energy literacy, proper socialization in this field is one of the most critical areas for emerging positive energy behaviors. This positive socialization requires positive behavioral patterns, cognition, and positive energy behavior. The best place to socialize is the family and the school, and these institutions can increase the energy literacy of citizens by teaching suitable behavioral patterns in the field of energy consumption.

Since this study only focused on the citizens of one city, it may be considered a limitation of the study. Therefore, future studies should include other cities in Iran and compare them; Because cultural diversity makes energy consumption behaviors in different cities diverse. However, a comparative study has not been possible due to the lack of research facilities. Another limitation of the research is not examining the relationship between demographic characteristics and energy literacy structures. In future research, the relationship between variables such as age, gender, education, and other demographic variables with energy literacy structures should be tested, and these relationships should be explained.

## Declarations

### Author contribution statement

Hamed Sayarkhalaj: Conceived and designed the experiments; Performed the experiments; Analyzed and interpreted the data; Contributed reagents, materials, analysis tools or data; Wrote the paper.

Majid Fatemi Khesal: Conceived and designed the experiments; Performed the experiments; Contributed reagents, materials, analysis tools or data; Wrote the paper.

### Funding statement

This research did not receive any specific grant from funding agencies in the public, commercial, or not-for-profit sectors.

### Data availability statement

Data will be made available on request.

### Declaration of interest’s statement

The authors declare no conflict of interest.

### Additional information

No additional information is available for this paper.
